# An overview of gambling disorder: from treatment approaches to risk factors

**DOI:** 10.12688/f1000research.12784.1

**Published:** 2018-04-09

**Authors:** José M Menchon, Gemma Mestre-Bach, Trevor Steward, Fernando Fernández-Aranda, Susana Jiménez-Murcia

**Affiliations:** 1 Department of Psychiatry, Bellvitge University Hospital-IDIBELL, Barcelona, Spain; 2 Departament of Clinical Sciences, School of Medicine, University of Barcelona , Barcelona, Spain; 3 CIBER Salud Mental (CIBERSAM), Instituto Carlos III, Barcelona, Spain; 4 Ciber Fisiopatología Obesidad y Nutrición (CIBERobn), Instituto de Salud Carlos III, Barcelona, Spain

**Keywords:** Gambling, addiction, Cognitive behaviour

## Abstract

Gambling disorder (GD) has been reclassified recently into the “Substance-Related and Addictive Disorders” category of the *Diagnostic and Statistical Manual of Mental Disorders, Fifth Edition* (DSM-5), a landmark occurrence for a behavioral addiction. GD is characterized by recurrent, maladaptive gambling behavior that results in clinically significant distress. Although the number of randomized controlled trials assessing the effectiveness of pharmacological treatments is limited, some pharmacological treatments, notably opiate antagonists, have been employed in the treatment of GD. Patients with GD often present cognitive distortions and specific personality traits, making treatment more difficult. Cognitive behavioral therapy has become the most common psychological intervention for treating gambling problems, and it is effective in reducing gambling behavior. In this brief overview, we provide a report on the state of pharmacological and psychological treatments for gambling disorder. Risk factors and potential future lines of research are addressed.

## Introduction

Gambling disorder (GD) is a psychiatric condition featuring recurrent, maladaptive gambling behavior that leads to clinically significant distress. GD was reclassified recently into the “Substance-Related and Addictive Disorders” group of the *Diagnostic and Statistical Manual of Mental Disorders, Fifth Edition* (DSM-5) ^1^, a first for a behavioral addiction. The recategorization of GD was essentially due to the similarities between this clinical condition and substance use disorders. Numerous studies find analogous characteristics between the two in reference to diagnostic criteria, symptomatology, genetic vulnerabilities, high rates of comorbidity, and their association with biological markers and cognitive deficits ^2, 3^. Furthermore, considering GD a behavioral addiction raises issues regarding the perceived dangerousness of the disorder as well as attitudes toward the chances of recovery and responsibility for creating and solving gambling-related problems ^4^.

Although gambling represents a harmless activity for most people who gamble, patients with GD are often characterized by cognitive distortions, such as illusions of control, impulsive behavior, and dysfunctional personality traits (for example, high harm avoidance or high novelty seeking) ^5^. Cognitive behavioral therapy (CBT) has become the most common psychological intervention for treating GD and has been demonstrated to be effective in reducing problem gambling behavior ^6– 9^. Pharmacological treatments have also been employed in the treatment of GD, although the number of randomized controlled trials assessing the effectiveness of these interventions is limited ^10– 12^. Several risk factors for developing GD have been identified, and prevention/harm-reduction efforts have provided mixed results ^13, 14^. In this brief review, we will aim to provide a report on the state of the art of pharmacological and psychological treatments for GD. Risk factors for GD will also be covered, and potential future lines of research will be addressed.

## Psychological treatment approaches

Despite pharmacological options to palliate GD symptomatology, several reviews of the literature point to psychological treatments as the most effective option for this disorder, and these are associated with significant improvements in both the short and the long term ^15, 16^.

Recent findings on different therapeutic approaches for GD will be presented in this review. However, it should be noted that, despite the relevant research advances in psychiatric disorder management, the understanding of treatment options for GD remains limited ^17^.

### Motivational interviewing

One of the most promising therapeutic options for GD is the motivational interview, either as a single treatment ^18– 20^ or in combination with other techniques ^21, 22^. This directive intervention empowers patients to identify and effectively solve their ambivalence about change ^19^. One of the central elements of this approach is normative feedback. Through this technique, individuals analyze their problematic gambling behavior, which is usually underestimated, comparing it with gambling patterns of the general population in order to promote a behavioral change ^23^. Different studies have reported that this therapeutic intervention is associated with a reduction of gambling behavior frequency and the severity of the disorder ^20^ and that these clinical changes remain present during the follow-up period ^19^. Likewise, other studies have observed an improvement in psychosocial functioning and the quality of life of these patients ^21^.

### Cognitive behavioral therapy

CBT has been shown to be especially effective for this behavioral addiction ^16, 17, 24^. Literature in this field stresses the importance of including motivational components ^16^ and cognitive restructuration ^9, 25^ in CBT programs in order to facilitate patients’ understanding of cognitive distortions related to gambling behavior and to weaken, among other factors, perseveration patterns, irrational beliefs, and magical thinking associated with this disorder ^7^.

Despite the effectiveness of CBT, few people with gambling problems seek clinical help ^26^, and this has led to an increase in research focusing on barriers that interfere with treatment access, such as lack of knowledge about treatment options or fear of stigma associated with the diagnosis of a psychiatric disorder, among many other factors ^26– 28^.

### Alternative approaches to enhance cognitive behavioral therapy

Owing to the complexity of GD and CBT limitations, unifying different approaches in order to enhance their effectiveness—instead of focusing on selecting only one clinical option—has been considered by the medical community in recent years ^9^. Some of the CBT limitations are high dropout and relapse rates during treatment ^6, 29– 32^, low compliance with therapeutic guidelines, specific personality traits such as novelty seeking and impulsivity, and deficits in emotion regulation ^33– 35^. On the other hand, these underlying factors may be more difficult to modify through standard CBT ^36, 37^.

Furthermore, GD heterogeneity must be taken into account when assessing the most indicated treatment ^38^. From an ecological perspective, several studies have demonstrated that GD is a complex disease in which diverse neurobiological and psychosocial vulnerability factors interact among them. Some approaches have tried to identify more homogeneous subgroups, which may share phenotypic and even endophenotypic characteristics. In general terms, three subgroups have been described—behaviorally conditioned, emotionally vulnerable, and antisocial impulsive ^39, 40^—both in community populations ^41, 42^ and in clinical samples ^38^. Moreover, these subgroups have been able to be replicated in populations of adolescents and young people ^43, 44^. In this vein, it is essential to have different therapeutic options that fit with the type of problem gambling behavior of each patient as well as other relevant clinical, psychopathological, and personality features ^5, 31, 45^.

On the one hand, a recently proposed option has been telephone interventions ^46^ and Internet-based CBT interventions, which may present positive aspects for patients with GD, such as flexibility, anonymity, and confidentiality ^47– 49^. Likewise, this type of approach has shown satisfactory results in the reduction of the severity of gambling problems as well as in the levels of anxiety, depression, and quality of life, both at the end of the treatment and in the follow-up at 36 months ^50, 51^. Promising studies indicate that Internet-delivered CBT can be effective even for relatives of people with this disorder, decreasing their depressive and anxious symptoms ^52, 53^. However, these approaches are still under development, and empirical studies proving their effectiveness are required ^54^.

On the other hand, studies suggest that the practice of mindfulness, understood as a technique based on meditation and aimed at increasing the awareness of the present moment without judging it ^55^, has a significant impact on improving the affective state, reducing the levels of anxiety and perceived stress, and decreasing the experience of pain ^56, 57^. In the field of addictions, mindfulness has also shown positive effects, both in substance addiction and in GD, reducing the levels of severity, abstinence, and craving ^58– 61^ but also decreasing the psychological and emotional discomfort associated with the addictive behaviors ^62^. Even brief mindfulness intervention can decrease ruminations associated with gambling ^63^, increase cognitive and behavioral flexibility ^36^, and improve quality of life ^35^. Although the results obtained so far are promising, more research is needed in order to determine the exact role of these mechanisms in the GD treatment outcomes.

Similarly, the use of both virtual reality and serious video games allows the simulation of emotionally charged contexts in which patients with GD can apply the therapeutic tools they acquired through CBT ^35, 64^. Finally, the incorporation of concerned significant others in treatment programs, both offline and online, is becoming more commonplace after promising results in different studies ^53, 65^.

## Pharmacological treatment

Currently, there is no drug approved for GD, although clinical practice guidelines usually have a section on the use of psychopharmacology in the disorder. For instance, the guideline for GD published in 2011 in Australia ^66^ accepted that naltrexone could be employed to reduce gambling severity in people with gambling problems.

The efficacy and utility of a number of medications have been studied in GD. However, many studies are open trials or reports on single or several cases, and the number of randomized, double-blind, placebo-controlled trials has been scarcer. Nonetheless, several excellent reviews ^67– 69^ and meta-analyses ^12, 70^ on the use of psychopharmacological drugs in GD have been published.

Three main classes of pharmacological approaches have been used on the grounds of clinical characteristics and neuropharmacological action: antidepressants, opioid antagonists, and mood stabilizers. The use of these drugs has been supported by the relationships that may be considered with some groups of mental disorders, mainly (a) compulsive-impulsive disorders; (b) substance use disorders given that GD may be considered a behavioral addiction, which is the view assumed by DSM-5; or (c) bipolar disorder, which has clinical features similar to those of GD. From a neuropharmacological perspective, the drugs that have been studied have a pharmacological action on opioid, serotonergic, dopaminergic, or glutamatergic neurotransmitter pathways.

Antidepressants, particularly selective serotonin reuptake inhibitors (SSRIs), have been examined. However, only five randomized, double-blind, placebo-controlled trials with SSRIs (two with paroxetine ^71, 72^, two with fluvoxamine ^73, 74^, and one with sertraline ^75^) have been carried out, and only two SSRIs—paroxetine ^71^ and fluvoxamine ^73^—were shown to be significantly superior to placebo. Another study with bupropion, a dopamine and noradrenaline reuptake inhibitor, did not show significant differences with placebo ^76^.

### Opioid antagonists

Two out of four randomized, double-blind, placebo-controlled trials have found significant improvement with naltrexone compared with placebo, and positive results have been found in the two trials with nalmefene. A thorough meta-analysis concluded that opiate antagonists demonstrated a small but significant benefit compared with placebo ^70^. A more recent review ^11^ of the use of opioid antagonists on behavioral addictions concluded that both naltrexone and nalmefene were the only evidence-based pharmacological treatments for GD.

Either other drugs studied—particularly in randomized, double-blind, placebo-controlled trials—have shown negative results compared with placebo or the evidence is inconclusive. For instance, lithium has shown positive results in one trial on bipolar spectrum disorders. Other drugs modulating the glutamatergic pathway, such as topiramate, have yielded controversial results while N-acetylcysteine has shown positive results but only in a pilot study.

In summary, opiate antagonists are the drugs that have shown the most promising evidence as medications for GD. Overall, the results of the research on pharmacological treatments for GD show that there are few randomized, double-blind, placebo-controlled trials and that most of the studies are open trials with small sample sizes and scarce follow-up data.

## Risk factors

Adolescent gambling, despite being an illegal activity for minors to partake in, is relatively common ^77^. Studies have found that individuals under the age of 18 years often report taking part in a wide range of gambling activities, and young age is often reported as a common risk factor for developing GD ^78, 79^. A recent meta-analysis quantified the effect size of risk factors in GD. These include 13 individual risk factors (alcohol use frequency, antisocial behaviors, depression, male gender, cannabis use, illicit drug use, impulsivity, number of gambling activities, problem gambling severity, sensation seeking, tobacco use, violence, and under-controlled temperament), one relationship risk factor (peer antisocial behaviors), one community risk factor (poor academic performance), one individual protective factor (socio-economic status), and two relationship protective factors (parent supervision and social problems) ^80^.

Although the prevalence of GD is higher in younger age groups, it is also a considerable problem for many older adults. A recent meta-analysis found that older individuals with GD were more likely to be single or divorced/separated ^81^. In terms of reasons for engaging in gambling activities, their findings indicated that older adults gambled more in an effort to ameliorate negative emotional states because they may have limited access to other exciting activities or because they are unable to participate in activities that they were previously able to do and therefore they attempt to fill this gap with gambling. These factors, along with having a fixed income and limited prospects of future earnings, make them an extremely vulnerable group ^82^.

It should be noted that, in terms of other psychological risk factors, impulsivity is a common feature in nearly all addictions, including GD ^5, 83– 85^. Personality traits are associated with GD, yet no single profile can encompass all gamblers. However, there is a degree of consensus that harm avoidance, low self-directedness, and difficulties with decision making and planning are, alongside impulsivity and sensation seeking, closely associated with the risk of developing a gambling problem ^6^.

In comparison with the general population, individuals with GD have an increased risk for suicide. One study of treatment-seeking patients with GD reported that 32% of individuals had experienced suicidal ideation and that 17% had attempted suicide at least once ^86^, whereas another study found that 30.2% of patients reported one or more suicide attempts in the 12 months preceding GD treatment ^87^. Increased medical and psychiatric comorbidity leads to a significantly decreased quality of life because of GD, yet still only 10% of individuals with GD ever seek treatment for GD ^88^. However, some reports indicate that treatment-seeking rates are higher for patients with greater disorder severity ^89^.

## Conclusions

The present review provides an understanding of current attempts at developing more inclusive GD treatment approaches. Psychological and, more specifically, cognitive behavioral approaches have provided satisfactory results, at least in the short to medium term ^15, 90^. However, the combination of these programs with other therapeutic strategies, such as brief motivational interventions, mindfulness, or the use of new technologies, seems to be a promising approach in terms of cost-effectiveness. Likewise, innovation in the therapy of this disorder is important. Treatment studies suggest that a percentage of patients fail with the most traditional treatments; therefore, it is compulsory as clinicians and researchers to continue advancing in this field, informing patients of the potential risks and improving the results of the usual psychological therapies. On the other hand, from a pharmacological perspective, opiate antagonists have shown the most promising evidence as being effective medications for GD. Finally, in terms of outcome predictors, numerous individual and social risk factors have been identified in the scientific literature, and prevention efforts should be targeted to those most at risk ^80^.

Further studies are required that take differences in GD presentation into account in order to facilitate greater clinical applicability. Likewise, as suggested by some authors, psychiatric comorbidities, for example, are not usually included in studies, hindering the subsequent application of these therapeutic options to the clinical population ^9^. In addition, owing especially to the emergence of new platforms that facilitate gambling access, GD is characterized by high heterogeneity and their features are constantly changing. Having updated prevention and treatment plans which take these factors into account and fit the clinical characteristics of each patient is a challenge that should be considered in greater depth in future research.
